# Pre-operative anemia was associated with all-cause mortality in patients with vertebral fracture who underwent percutaneous vertebroplasty

**DOI:** 10.3389/fmed.2022.1058636

**Published:** 2022-11-21

**Authors:** Yun-Che Wu, Yu-Hsien Lin, Yu-Tsung Lin, Wen-Chien Wang, Kun-Hui Chen, Chien-Chou Pan, Jun-Sing Wang, Cheng-Hung Lee

**Affiliations:** ^1^Department of Orthopedics, Taichung Veterans General Hospital, Taichung, Taiwan; ^2^Department of Post-Baccalaureate Medicine, College of Medicine, National Chung Hsing University, Taichung, Taiwan; ^3^Department of Computer Science and Information Engineering, Providence University, Taichung, Taiwan; ^4^Department of Rehabilitation Science, Jenteh Junior College of Medicine, Nursing and Management, Miaoli, Taiwan; ^5^Division of Endocrinology and Metabolism, Department of Internal Medicine, Taichung Veterans General Hospital, Taichung, Taiwan; ^6^Ph.D. Program in Translational Medicine, National Chung Hsing University, Taichung, Taiwan; ^7^Department of Food Science and Technology, Hung Kuang University, Taichung, Taiwan

**Keywords:** anemia, fracture, mortality, vertebral fracture, vertebroplasty

## Abstract

**Purpose:**

We investigated the association between pre-operative anemia and long-term all-cause mortality in patients with vertebral fracture who underwent a vertebroplasty.

**Materials and methods:**

We retrospectively selected patients who were admitted for vertebroplasty for vertebral compression fracture between 2013 and 2020. Patients who had pathologic fractures or had no assessment of bone mineral density were excluded. Relevant information was collected from electronic medical records. Patients’ survival status was confirmed at the end of March 2021. Cox-proportional hazard models were conducted to examine the effects of anemia (<12 g/dL vs. ≥12 g/dL) and pre-operative hemoglobin levels (as a continuous variable) on all-cause mortality with multivariate adjustments.

**Results:**

A total of 167 patients were analyzed (mean age 75.8 ± 9.3 years, male 25.7%). After a median follow-up duration of 2.1 years, pre-operative anemia (hemoglobin <12 g/dL vs. ≥12 g/dL) was independently associated with a higher risk of all-cause mortality (hazard ratio 2.762, 95% CI 1.184 to 6.442, *p* = 0.019). An increase in pre-operative hemoglobin was associated with a lower risk of all-cause mortality after multivariate adjustment (hazard ratio 0.775, 95% CI 0.606 to 0.991, *p* = 0.042).

**Conclusion:**

Pre-operative anemia (<12 g/dL) was independently associated with survival outcome among patients with vertebral compression fractures who underwent vertebroplasty. Our findings highlight anemia as a risk factor of long-term mortality in this elderly surgical population.

## Introduction

Osteoporotic vertebral compression fracture is common in elderly populations (1, 2). Moreover, symptomatic vertebral compression fracture is associated with an increase in mortality risk (3) and an impairment of quality of life (4). These may result in substantial healthcare expenditures (5). Patients with persistent symptoms despite medical treatment are often treated with vertebroplasty, which has become a common orthopedic procedure. However, its effects on symptom relief, functional outcomes, and risk of mortality in the elderly are not yet clear (6–8).

Older aged individuals often have multiple morbidities which may further increase their susceptibility to post-operative complications and risk of long-term mortality. For example, anemia is prevalent in elderly people, especially hospitalized patients (9). Furthermore, anemia is an independent risk factor for all-cause mortality in people aged ≥65 years (10). Pre-operative anemia (defined as a hemoglobin <12 g/dL) has been associated with a higher risk of short-term mortality in patients with hip fracture (mortality within 60 days) (11) and in those who underwent primary total knee arthroplasty (mortality within 30 days) (12).

Given that anemia is frequently observed in elderly inpatients (9), and in light of the fact that patients who undergo vertebroplasty are often aged more than 70 years (13), pre-operative anemia might have an effect on long-term outcomes in this population. In this study, we investigated the association between pre-operative anemia and long-term all-cause mortality in patients with vertebral fracture who underwent vertebroplasty.

## Materials and methods

This study was conducted in accordance with the Declaration of Helsinki. The study protocol was approved by the Institutional Review Board of Taichung Veterans General Hospital, Taichung, Taiwan (approval number: CE22167A). We retrospectively selected patients who presented to our Department of Orthopedics with a vertebral compression fracture between 2013 and 2020. These patients were admitted for a vertebroplasty after pre-operative assessment. We excluded patients who had pathologic fractures or history of blood transfusion within 3 months, and those with no assessment of bone mineral density from the analysis. Pathological fractures were confirmed by pathological reports and discharge diagnosis. The requirement for informed consent was waived due to the retrospective study design.

We collected relevant information, including anthropometric data, medical history, and laboratory test results, from electronic medical records. Patients’ survival status was confirmed at the end of March 2021 using data obtained from the Ministry of Health and Welfare, R.O.C. Thereafter, de-identified data were used for analyses. Hemoglobin levels were measured as a routine pre-operative assessment. The study population was divided into two groups according to their pre-operative hemoglobin levels (<12 g/dL vs. ≥12 g/dL) to examine the association between pre-operative anemia and long-term all-cause mortality. The cutoff value 12 g/dL was determined because that it is the lower limit of normal, and a hemoglobin level less than 12 g/dL has been associated with risk of mortality in patients receiving an orthopedic surgery (11, 12, 14).

### Statistical analysis

We conducted the statistical analyses using the Statistical Package for the Social Sciences (IBM SPSS version 22.0; International Business Machines Corp., NY, USA). Categorical and continuous variables were examined for statistically significant between-group differences using the chi-square test and independent *t*-test, respectively. Kaplan–Meier survival curves were plotted for the study population according to their pre-operative hemoglobin levels (<12 g/dL vs. ≥12 g/dL). Cox-proportional hazard models were conducted to examine the effects of anemia (<12 g/dL vs. ≥12 g/dL) and pre-operative hemoglobin levels (as a continuous variable) on all-cause mortality with adjustments for age, sex, and concomitant chronic diseases. Cubic spline of pre-operative hemoglobin levels vs. risk of all-cause mortality by a Cox proportional hazards model was conducted as a sensitivity test. A two-sided *p*-value of less than 0.05 was considered statistical significance.

## Results

A total of 167 patients were analyzed (mean age 75.8 ± 9.3 years, male 25.7%). Table 1 shows the characteristics of the study population according to their pre-operative hemoglobin levels (<12 g/dL vs. ≥12 g/dL). Patients who had a pre-operative hemoglobin <12 g/dL (mean 10.4 ± 1.0 g/dL) had a lower body mass index (23.0 ± 4.3 vs. 24.6 ± 3.9 kg/m^2^, *p* = 0.014), and a higher proportion of diabetes (30.2 vs. 13.5%, *p* = 0.009) and chronic kidney disease (52.4 vs. 23.1%, *p* < 0.001), compared with those who had a pre-operative hemoglobin ≥12 g/dL (mean 13.4 ± 1.0 g/dL). There were no significant between-group differences in the other variables.

**TABLE 1:** Baseline characteristics of the study population according to pre-operative hemoglobin.

Variables	<12 g/dL	≥12 g/dL	*P*-value
*N*	63	104	
Age, years	76.7 ± 9.8	75.2 ± 9.0	0.314
Male sex, *n* (%)	16 (25.4)	27 (26.0)	0.936
Body mass index, kg/m^2^	23.0 ± 4.3	24.6 ± 3.9	0.014
Smoking, *n* (%)	4 (6.3)	8 (7.7)	0.745
Diabetes, *n* (%)	19 (30.2)	14 (13.5)	0.009
Hypertension, *n* (%)	38 (60.3)	51 (49.0)	0.157
Chronic kidney disease, *n* (%)	33 (52.4)	24 (23.1)	< 0.001
Hemoglobin, g/dL	10.4 ± 1.0	13.4 ± 1.0	< 0.001
Osteoporosis, *n* (%)	43 (68.3)	81 (77.9)	0.168
Medication for osteoporosis, *n* (%)^a^	38 (60.3)	68 (65.4)	0.510
Level of vertebroplasty, *n* (%)			0.122
T-spine	22 (34.9)	49 (47.1)	
L-spine	41 (65.1)	55 (52.9)	

Values are mean ± SD or n (%).

^a^Bisphosphonate, receptor activator of nuclear factor kappa-B inhibitor, or parathyroid hormone.

**FIGURE 1:**
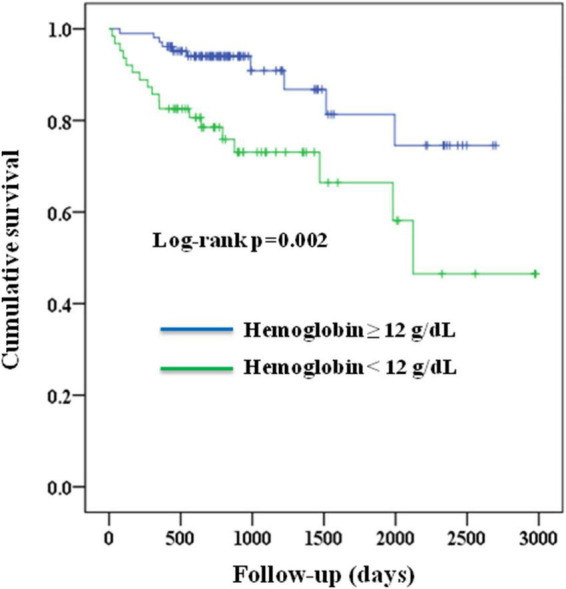
Kaplan–Meier survival curves of the study population according to their pre-operative hemoglobin levels (<12 g/dL vs. ≥12 g/dL).

After a median follow-up duration of 2.1 years, patients with a pre-operative hemoglobin <12 g/dL had a lower survival rate than those who had a pre-operative hemoglobin ≥12 g/dL (Figure 1, Log-rank *p* = 0.002). The associations of pre-operative anemia (hemoglobin <12 g/dL vs. ≥12 g/dL) and hemoglobin levels with all-cause mortality are shown in Table 2. Pre-operative anemia (hemoglobin <12 g/dL vs. ≥12 g/dL) was associated with a significantly higher risk of all-cause mortality (hazard ratio 3.164, 95% CI 1.459 to 6.860, *p* = 0.004). This association remained significant after multivariate adjustment (hazard ratio 2.762, 95% CI 1.184 to 6.442, *p* = 0.019).

**TABLE 2:** Associations of pre-operative anemia and hemoglobin with all-cause mortality.

Independent variable	Hazard ratio (95% CI)	*P*
**Anemia (hemoglobin <12 g/dL vs. ≥12 g/dL)**		
Model 1	3.164 (1.459, 6.860)	0.004
Model 2	2.986 (1.371, 6.501)	0.006
Model 3	2.762 (1.184, 6.442)	0.019
**Hemoglobin (g/dL)**		
Model 1	0.709 (0.574, 0.876)	0.001
Model 2	0.733 (0.591, 0.909)	0.005
Model 3	0.775 (0.606, 0.991)	0.042

Model 1, unadjusted. Model 2, adjusted for age and sex. Model 3, adjusted for variables in Model 2 plus body mass index, smoking, diabetes, hypertension, chronic kidney disease, osteoporosis, and medical treatment for osteoporosis.

**FIGURE 2:**
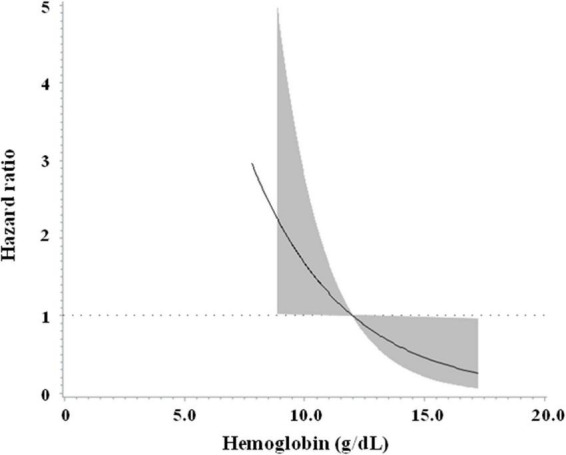
Cubic spline of pre-operative hemoglobin levels vs. risk of all-cause mortality in the overall population.

Similar findings were noted when pre-operative hemoglobin levels were examined as a continuous variable. An increase in pre-operative hemoglobin was associated with a lower risk of all-cause mortality (hazard ratio 0.709, 95% CI 0.574 to 0.876, *p* = 0.001, Table 2). This association was independent of age, sex, body mass index, and other clinical variables (hazard ratio 0.775, 95% CI 0.606 to 0.991, *p* = 0.042). Figure 2 shows the cubic spline of pre-operative hemoglobin levels vs. risk of all-cause mortality in the overall population. The point of hemoglobin level below which the risk of mortality started to increase was approximately 12 g/dL.

## Discussion

In this study, we demonstrated that pre-operative anemia (hemoglobin <12 g/dL) was associated with a higher risk of all-cause mortality in patients with vertebral compression fracture who underwent percutaneous vertebroplasty after a median follow-up duration of more than 2 years. The findings were consistent when pre-operative hemoglobin was examined as an independent continuous variable (Table 2). Our results highlight the importance of pre-operative anemia as a risk factor for long-term mortality in patients with vertebral compression fracture who underwent a surgical intervention.

The incidence of vertebral fracture substantially increases in an aging population (15, 16). Furthermore, there is a significant increase in mortality rate after vertebral fracture (16, 17). In a recent report (18), anemia was associated with an approximately twofold increased risk of osteoporotic fractures in elderly men. Hence, anemia may be associated with adverse outcomes in that population (19, 20). In this context, anemia has been associated with complications after some orthopedic surgeries (21, 22). Moreover, a low pre-operative hemoglobin level was associated with an increase in in-hospital and 30-days mortality after surgical procedures (23). Similar findings were noted in patients who underwent total knee arthroplasty (12) and in those with hip fractures (11). However, data on the association between pre-operative anemia and long-term mortality after orthopedic procedures are scarce (14). Since the prevalence of anemia may be as high as 40% in elderly inpatients (9), our findings are important and could be used to identify patients who are at a high risk of post-operative long-term mortality in those who underwent percutaneous vertebroplasty (24–26), which has become a common orthopedic procedure for treatment of compression vertebral fractures.

The mechanisms by which pre-operative anemia was related to risk of mortality are not yet clear. In a prospective cohort study (14), pre-operative anemia (<12 g/dL) was associated with a higher risk of mortality (adjusted relative risk 1.68, 95% CI 1.22 to 2.30, *p* = 0.001) in elderly people with hip fracture. Anemia may impede functional mobility (27, 28) after surgery for fracture in the elderly, which could be associated with subsequent complications (29–32), such as pneumonia, and deep vein thrombosis. Anemia is frequently observed in aging populations. In light of this phenomenon, our findings highlight the effects of pre-operative anemia on adverse outcomes (33) in patients who underwent vertebroplasty, which is commonly conducted in elderly patients with vertebral fracture. The same scenario should be carefully considered in patients undergoing other orthopedic surgeries. In a retrospective cohort study including patients with femoral neck fracture aged 65 years and above treated with hemi-arthroplasty (34), pre-operative anemia (on-admission hemoglobin <10 g/dL) was associated with a higher risk of 2-years mortality (hazard ratio 3.3, 95% CI 1.3 to 8.3, *p* < 0.01). Similar findings were noted in another study conducted in patients over 60 years old who underwent hip fracture surgery (35). These results are in line with a recent report (36) in which hemoglobin was selected as a predictor for 1-year post-operative mortality in geriatric patients with hip fractures using an artificial intelligence approach. According to the aforementioned findings, pre-operative anemia should be evaluated carefully in aging surgical populations. These patients’ outcomes might be improved after optimal diagnosis and management of anemia.

It is not clear if various causes of anemia would affect the association between pre-operative anemia and patients outcomes. Although we did not have differential diagnosis of anemia for all patients, some of them (*n* = 132, 79%) had data on mean corpuscular volume (MCV). Among these, 17 (12.9%) had an MCV < 80 fL and 17 (12.9%) had an MCV > 100 fL. The association between anemia and all-cause mortality remained significant (HR 2.806, 95% CI 1.132 to 6.957, *p* = 0.026) after adjustment for MCV in the multivariate analysis. Similar finding was noted when hemoglobin was analyzed as a continuous variable (HR 0.772, 95% CI 0.598 to 0.996, *p* = 0.047). Based on these findings, we consider that the causes of anemia might be unlikely to affect the association between anemia and long-term all-cause mortality in our study population.

The strength of this study is that the association between pre-operative anemia and mortality after vertebroplasty was examined with a median follow-up duration of more than 2 years. Nevertheless, there are several limitations. First, the number of our patients was relatively small. Second, the data were retrospectively collected in a single medical center. Although we had adjusted for some concomitant chronic diseases (such as hypertension and diabetes) in the analytical models, missing data on the status of disease control (e.g., blood pressure and blood glucose levels) and nutrition might have confounded our results. Third, the effect of a blood transfusion on the association between pre-operative anemia and long-term mortality risk was not examined. Some of the aforementioned issues need to be addressed in prospective studies.

In conclusion, pre-operative anemia (<12 g/dL) was independently associated with survival outcome among patients with vertebral compression fractures who underwent vertebroplasty. Our findings highlight anemia as a risk factor for long-term mortality in this aging surgical population.

## Data availability statement

The datasets presented in this article are not readily available because privacy/ethical restrictions. Requests to access the datasets should be directed to J-SW, jswang@vghtc.gov.tw.

## Ethics statement

The studies involving human participants were reviewed and approved by the Institutional Review Board of Taichung Veterans General Hospital, Taichung, Taiwan. Written informed consent for participation was not required for this study in accordance with the national legislation and the institutional requirements.

## Author contributions

Y-CW, J-SW, and C-HL designed and conducted the research. Y-CW, Y-HL, Y-TL, W-CW, K-HC, C-CP, and C-HL contributed to data curation. Y-CW and J-SW analyzed data and wrote the first draft of the manuscript. Y-HL, Y-TL, W-CW, K-HC, C-CP, and C-HL revised the manuscript critically for important intellectual content. All authors approved the final draft of the manuscript.
